# Methicillin-resistant Staphylococcus aureus in Metabolic Syndrome Patients at the Mbouda Hospitals, West Region of Cameroon

**DOI:** 10.7759/cureus.7274

**Published:** 2020-03-15

**Authors:** Wiliane Jean Takougoum Marbou, Victor Kuete

**Affiliations:** 1 Biochemistry, Faculty of Sciences, University of Dschang, Dschang, CMR; 2 Biochemistry, University of Dschang, Dschang, CMR

**Keywords:** staphylococcus aureus, intestinal carriage, oxacillin resistance, metabolic syndrome, mbouda-cameroon

## Abstract

Background

Studies have revealed an increased risk of contracting S*taphylococcus aureus* infections in patients suffering from metabolic diseases. Methicillin-resistant *Staphylococcus aureus* (MRSA) in metabolic syndrome subjects is less reported in the medical literature. This study aimed at isolating and establishing the distribution of antibiotic-resistant *Staphylococcus aureus* from faecal samples in metabolic syndrome subjects from Mbouda Hospitals, West Region of Cameroon.

Methods

A cross-sectional study was conducted from May 2016 to May 2018 in 114 participants in whom *Staphylococcus aureus* was detected. Thirty (30) participants were suffering from metabolic syndrome and 84 did not suffer from this pathology. *Staphylococcus aureus* isolation was based on culture and confirmed by polymerase chain reaction (PCR) of the *nuc* gene. The Kirby-Bauer disk diffusion method was used for drug susceptibility assay. Molecular detection of the *mecA* gene by PCR was performed to screen MRSA.

Results

From the 114 *Staphylococcus aureus* isolates, the prevalence of the *mecA* gene confirming MRSA was 79.82%, higher than that of methicillin-sensitive *Staphylococcus aureus (MSSA)* (20.17%). The frequency of MRSA was higher in participants with metabolic syndrome (80.00%) compared to non-metabolic syndrome (79.76%) participants without significant difference (p=0.977). The antimicrobial susceptibility test revealed that the amikacin susceptibility profile was significantly different in metabolic and non-metabolic syndrome participants (p=0.037, chi-square=6.59). Regarding metabolic syndrome status, 72.62% of isolates were multidrug-resistant in non-metabolic syndrome participants versus 63.33% in metabolic syndrome participants.

Conclusion

This study suggests that metabolic syndrome patients harbour MRSA strains in their intestines even as the difference was not statistically significant with non-metabolic syndrome participants. The need for appropriate antimicrobial use to halt or at least limit the spread of resistance is suggested in the care of metabolic syndrome patients and the entire population.

## Introduction

Some studies have revealed that there is an increased risk of contracting *Staphylococcus aureus* infections in patients with diabetes and obesity than in those without [1]. Obesity and type 2 diabetes are recognized as a determining factor in the development of metabolic syndrome [2]. Methicillin-resistant *Staphylococcus aureus* epidemiology in the metabolic syndrome patients is less known. *Staphylococcus aureus* is an important opportunistic pathogen responsible for a variety of diseases. It is a gram-positive bacterium that occurs naturally in and on the human body. *Staphylococcus aureus* is found in the axillae (8%), chest/abdomen (15%), perineum (22%), and intestine (17-31%) [3]. In healthy individuals, an intestinal carriage of 20% for Staphylococcus aureus has been reported and although nasal carriage may predispose them to intestinal carriage, sole intestinal carriage was also detected [4]. From the nasopharynx, the bacterium is propagated on the skin by aerosol and is often present on clothes and dander [4]. As *staphylococci* are resistant to desiccation, transmission can not only be direct, through the hands of health care workers in hospitals but also indirectly by objects and dust [5]. These several niches in the body form major reservoirs for the *Staphylococcus aureus* infection. *Staphylococcus aureus* colonisation is a major risk factor for staphylococcal infections.

The human intestinal tract harbours a large number of cultivable and non-cultivable bacteria. The colonization of the gastrointestinal tract by *Staphylococcus aureus* has been documented as potential sources of both endogenous and exogenous staphylococcal infections [6-7]. Stools specimens can surely be significant as a source of environmental contamination and have been identified as a possible source of antibiotic-resistant *Staphylococcus aureus* especially methicillin-resistant *Staphylococcus aureus* (MRSA).

MRSA is becoming more of a public health concern. MRSA causes skin infections, such as paronychia, styles, and furunculosis and life-threatening systemic infections such as sepsis, pneumonia, and endocarditis [8]. The resistance of *Staphylococcus aureus* to methicillin is caused by a gene name *mecA*, which alters the site at which methicillin binds to kill the organism. *Staphylococcus aureus* bacteria resistant to methicillin are also resistant to many other antibiotics because the same cellular process is used by bacteria to be resistant to other antibiotics [9].

To the very best of our knowledge, very few studies have focused on the intestinal reservoir of MRSA. Similarly, no comprehensive studies regarding the distribution of antibiotic-resistant *Staphylococcus aureus* from faecal samples in metabolic syndrome subjects have been reported in the medical literature. In this study, we presumed that the faeces of metabolic syndrome subjects might contain MRSA strains and would have the possibility to serve as a potential source of MRSA dissemination in the Bamboutos Division, West Region of Cameroon. The aim of this study, therefore, was to determine the distribution of the antibiotic-resistant *Staphylococcus aureus* from faecal samples in metabolic syndrome subjects from Mbouda Hospitals, West Region of Cameroon.

## Materials and methods

Subjects and study design

This study was conducted in a sub-sample of the study carried out to estimate the prevalence of metabolic syndrome and its components in Bamboutos Division’s adults (20 years old and older), West Region of Cameroon [10]. One hundred and fourteen (114) patients from whom the presence of *Staphylococcus aureus* was detected in the faeces were included in this study. Four-hundred ninety (490) were excluded. This was a cross-sectional study conducted from May 2016 to May 2018. Patients coming for consultation for enteric disorders at the Mbouda AD LUCEM and District Hospitals, two reference hospitals in Bamboutos Division, were recruited. Human immunodeficiency virus (HIV) positive patients, pregnant women, patients under antibiotic treatment, and participants with positive serology for Hepatitis B and C were not included in this study.

Data collection

Data collection and examination was performed as part of the study previously described by Marbou and Kuete (2019) [10]. Briefly, they comprised standardized questionnaire, clinical examinations with blood pressure at rest (OMROM 705), fasting blood glucose (ACCU-CHEK, Mannheim, Germany), lipid profile (MaestroNano, MaestroGen, USA), waist circumference measurement and stool collection. Metabolic syndrome was defined using the Joint Interim Statement of the International Diabetes Federation Task Force on Epidemiology and Prevention definition [11].

Stools collection

Six hundred and four (604) stool specimens were collected using sterile bottles (Viamed, Miami Lakes, Florida). The specimens collected were first inoculated in nutrient agar (NA) (HiMedia, Mumbai, India), sub-cultured in mannitol salt agar (MSA) (Uptima/Interchim, France), and aerobically incubated for 24 hours at 37°C. Bacterial colonies showing the typical characteristics of *Staphylococcus aureus,* including golden-yellow colour colonies on MSA, were subjected to molecular confirmation by polymerase chain reaction (PCR).

Molecular confirmation of *Staphylococcus aureus*

In this study, the TENT (Tris-EDTANaCl-TritonX100) method for Staphylococcus aureus deoxyribonucleic acid (DNA) extraction was used as described by Hassanzadeh et al. (2016) [12]. *Staphylococcus aureus* was confirmed by PCR of the *nuc* gene as previously described by Brakstad et al. (1992) using primers listed in Table 1 [13].

**Table 1:** Polymerase chain reaction (PCR) primers and conditions used in this study [F]: Forward primer; [R]: Reverse primer; PCR: Polymerase chain reaction

Reference	Nucleotide sequences of primer [primer]	Target	PCR conditions			Size of amplicon (bp)
			Denaturing	Annealing	Extension	
[13]	5′GCGATTGATGGTGATACGGTT-3′ [F] 5′-AGCCAAGCCTTGACGAACTAAAGC-3′ [R]	nuc	94°C, 60s	55°C, 30s	72°C, 90s	267
[14]	5’- AAA ATC GAT GGTAAA GGTTGG C - 3’ [F] 5’- AGTTCTGCAGTACCG GAT TTG C-3’ [R]	mecA	94°C, 60s	55°C, 30s	72°C, 60s	532

Antimicrobial susceptibility testing

The Kirby-Bauer disk diffusion method was performed as per the recommendations of the Clinical and Laboratory Standards Institute (CLSI) guidelines [14]. A bacterial suspension equivalent to the 0.5 McFarland turbidity standard was prepared and inoculated on Mueller-Hinton agar (MHA) (Accumix, Mol, Belgium) supplemented with 2% NaCl. Standard antimicrobial disks representing multiple drug classes were subsequently set for oxacillin (beta-lactam) (1 µg), gentamicin (aminoglycosides) (10 µg), amikacin (aminoglycosides) (30 µg), chloramphenicol (chloramphenicol) (30 µg), doxycycline (tetracycline) (30 µg), and co-trimoxazole (sulfonamides) (23.75/1.25 µg) (Becton Dickinson and Company, Sparks, Maryland). The plates were incubated at 37°C for 24 hours. An inhibition zone diameter of each antimicrobial was then measured and interpreted as resistant (R), intermediate (I), and sensitive (S). mecA-negative *Staphylococcus aureus* ATCC 29213 and *mecA*-positive *Staphylococcus aureus* ATCC 33591 were used as the control in each test run. Inhibition zones were interpreted according to CLSI guidelines [14].

Molecular detection of the *mecA* gene by PCR

The presence of the *mecA* gene responsible for the resistance of *Staphylococcus aureus* to methicillin was detected by PCR in all confirmed *Staphylococcus aureus* resistant to Oxacillin. *mecA* was amplified using specifics *mecA* primers (Table 1) as described by Kumurya, Gwarzo, and Uba (2015) [15]. Amplification was done in a PCR solution of 20 µl containing 11.25 µl nuclease-free water, 2.5 µl of 10xPRC buffer (New England Biolab, Hitchin, UK), 2.0 µl dNTP Mix (Thermo Fischer Scientific, UK), 1 μl of forward and reverse primer each, 2 μl of DNA template, and 0.25 μl of Taq DNA polymerase (New England Biolab, Hitchin, UK). DNA amplification was carried out in a Techne PCR system TC-5000 thermocycler (Bibby Scientific Ltd., Essex, UK) with the following thermal-cycling profile: an initial denaturation step at 94°C for five min, followed by one cycle of amplification starting by denaturation at 94°C for 30 s, annealing at 55°C for 30 s, and extension at 72°C for 60 s, ending with a final extension step at 72°C for five min. mecA-positive *Staphylococcus aureus* ATCC 33591 was used and PCR products were visualized after electrophoresis on 2% agarose gel stained with ethidium bromide.

Ethical consideration

Participants received oral and written information about the study and signed an informed consent form. The protocol was approved by the Cameroon National Ethics Committee (CNEC), Ministry of Public Health (reference number, 2018/06/ 1054/CE/CNERSH). The study was conducted in agreement with the Declaration of Helsinki.

Statistical analysis

The data collected were analyzed by Epi Info™ version 7.2.2.6 (CDC, 1600 Clifton Road, Atlanta). The chi-square (χ2) test was used to evaluate the relationship between antimicrobial resistance and specific variables. A p-value of <0.05 was considered statistically significant.

## Results

**Figure 1:**
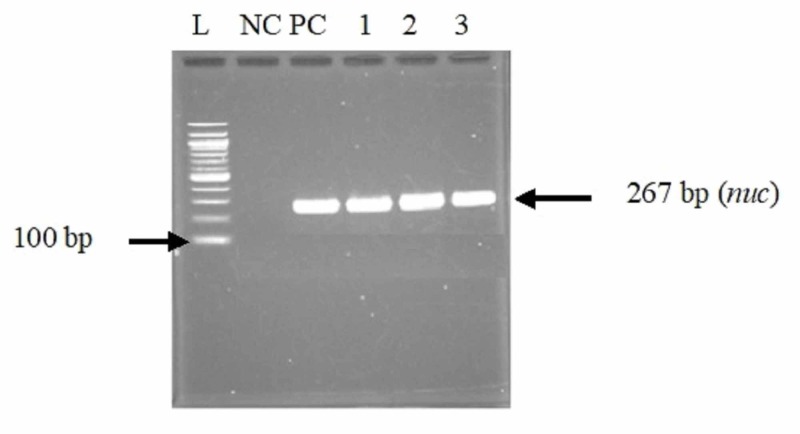
Gel electrophoresis profile of the nuc gene for the confirmation of Staphylococcus aureus isolates Lane L: molecular size marker (100 bp DNA ladder); Lane NC: negative control; Lane PC: positive control; Lanes 1, 2, and 3: positive Staphylococcus aureus DNA: deoxyribonucleic acid

**Figure 2:**
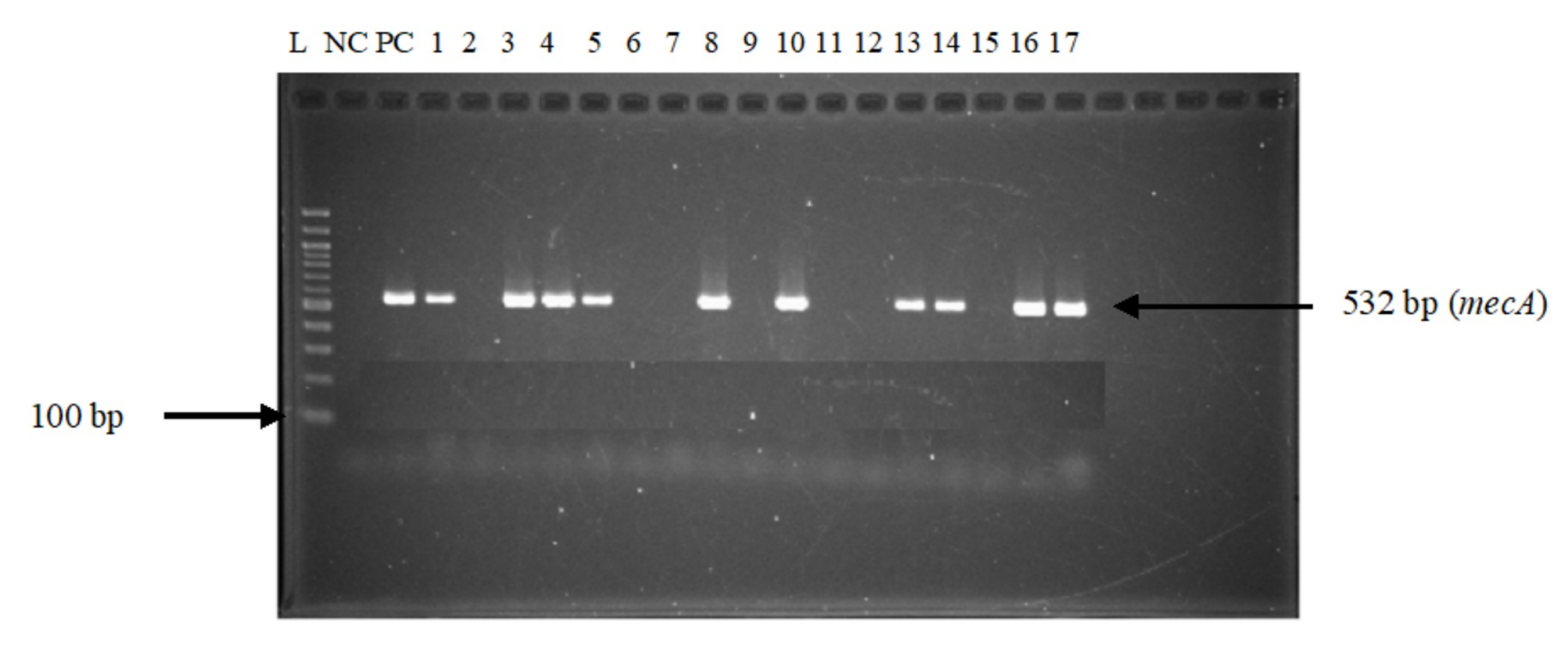
Gel electrophoresis profile of the mecA gene for the detection of methicillin-resistant Staphylococcus aureus Lane L: molecular size marker (100 bp DNA ladder); Lane NC: negative control; Lane PC: positive control; Lanes 1, 3, 4, 5, 8, 10, 13, 14, 16, and 17: methicillin-resistant Staphylococcus aureus; Lanes 2, 6, 7, 11, 12 and 15: methicillin-sensitive Staphylococcus aureus DNA: deoxyribonucleic acid

A total of 114 participants from whom the *Staphylococcus aureus* was isolated, and confirmed by PCR (using the *nuc* gene) (Figure 1), in the faeces were studied. Thirty (30) participants were suffering for metabolic and 84 did not suffer from this pathology. The presence of the *mecA* gene responsible for the resistance of *Staphylococcus aureus* to methicillin was detected by PCR in all confirmed Staphylococcus aureus (Figure 2). The prevalence of the *mecA* gene confirming MRSA was 79.82% (n=91).

**Figure 3:**
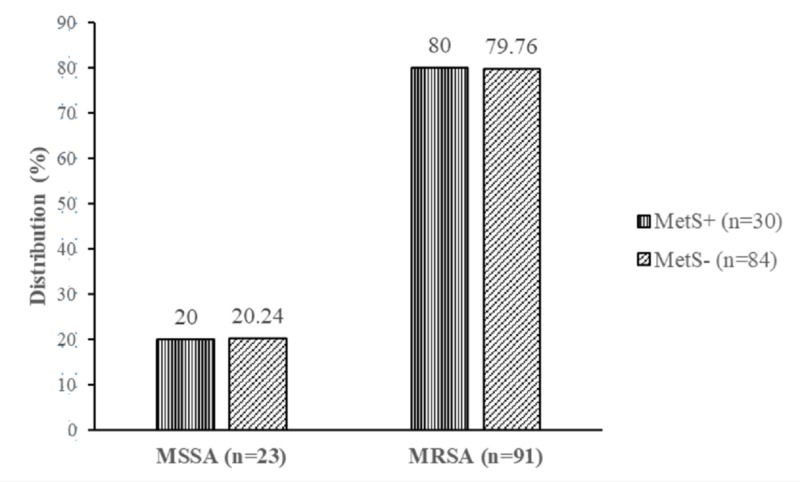
Distribution of methicillin-sensitive and resistant Staphylococcus aureus according to metabolic syndrome status MSSA: methicillin-sensitive Staphylococcus aureus; MRSA: methicillin-resistant Staphylococcus aureus; MetS: metabolic syndrome

The age range of the study participants was from 20-83 years. The frequency of MRSA was higher in participants with metabolic syndrome (80.00%) as compared to non-metabolic syndrome (79.76%) participants without a significant difference (p=0.977) (Figure 3).

The distribution of *Staphylococcus aureus* among participants with regard to gender and age was studied. Most of the Staphylococcus aureus were isolated in the age groups of 20 to <30 years. In the 114 *Staphylococcus aureus* isolates, the frequency of MRSA 91 (79.83%) was higher than that of MSSA 23 (20.17%). The frequency of MRSA in males was 41 (80.39%) and that of females was 50 (79.37%). A high frequency of MRSA was observed in participants aged 60 to <70 and ≥70 years (92.86% and 100.00), respectively, in which a lower incidence of MSSA was observed (7.14% and 0.00%), respectively (Table 2).

**Table 2:** Faecal carriage of Staphylococcus aureus among participants with regard to gender and age MSSA: methicillin-sensitive Staphylococcus aureus; MRSA: methicillin-resistant Staphylococcus aureus; MetS: metabolic syndrome

Variables		Presence of Staphylococcus aureus		Total (N)
		MSSA (N (%))	MRSA (N (%))	
Sex	Male	10 (19.61)	41 (80.39)	51
	Female	13 (20.63)	50 (79.37)	63
Age group (year)	20 - <30	11 (35.48)	20 (64.52)	31
	30 - <40	1 (11.11)	8 (88.89)	9
	40 - <50	7 (25.93)	20 (74.07)	27
	50 - <60	3 (15.79)	16 (84.21)	19
	60 - <70	1 (7.14)	13 (92.86)	14
	≥70	0 (0.00)	14 (100.00)	14

The overall susceptibility profiles of *Staphylococcus aureus* isolates are shown in Table 3. The antimicrobial susceptibility test revealed that the resistance of *Staphylococcus aureus* to oxacillin was higher in participants with metabolic syndrome than non-metabolic syndrome participants (100.00% and 92.86%) respectively, non-significant at p=0.322 (chi-square=2.26). Amikacin susceptibility profile was significantly different in metabolic and non-metabolic syndrome participants (p=0.037, chi-square=6.59). Eight (9.52%), 17 (20.24%) and 59 (70.24%) of *Staphylococcus aureus* were respectively resistant, Intermediate and sensitive to amikacin in metabolic syndrome versus 0 (0.00%), 12 (40.00%) and 18 (60.00%) in non-metabolic syndrome participants, respectively. In non-metabolic syndrome participants, doxycycline had the highest overall resistance of 85.71%, followed by co-trimoxazole (64.29%) and chloramphenicol (51.19%) while amikacin had the highest overall sensitivity of 70.24% followed by gentamicin (64.29%).

**Table 3:** Antimicrobial sensitivity pattern of Staphylococcus aureus strains to different antimicrobial agents with regard to MetS status a: non-metabolic syndrome participants; b: metabolic syndrome participants

Antibiotics	Resistant, N (%)	Intermediate, N (%)	Sensitive, N (%)	X^2^ (probability)
Oxacillin^a^	78 (92.86)	2 (2.38)	4 (4.76)	2.26 (0.322)
Oxacillin^b^	30 (100.00)	0 (0.00)	0 (0.00)	
Gentamicin^a^	20 (23.81)	10 (11.90)	54 (64.29)	0.99 (0.610)
Gentamicin^b^	6 (20.00)	2 (6.67)	22 (73.33)	
Amikacin^a^	8 (9.52)	17 (20.24)	59 (70.24)	6.59 (0.037)
Amikacin^b^	0 (0.00)	12 (40.00)	18 (60.00)	
Chloramphenicol^a^	43 (51.19)	1 (1.19)	40 (47.62)	0.99 (0.607)
Chloramphenicol^b^	13 (43.33)	0 (0.00)	17 (56.67)	
Doxycyclin^a^	72 (85.71)	7 (8.33)	5 (5.95)	1.81 (0.404)
Doxycycline^b^	3 (10.00)	23 (76.67)	4 (13.33)	
Co-trimoxazole^a^	54 (64.29)	12 (14.29)	18 (21.43)	0.54 (0.762)
Co-trimoxazole^b^	18 (60.00)	6 (20.00)	6 (20.00)	

**Figure 4:**
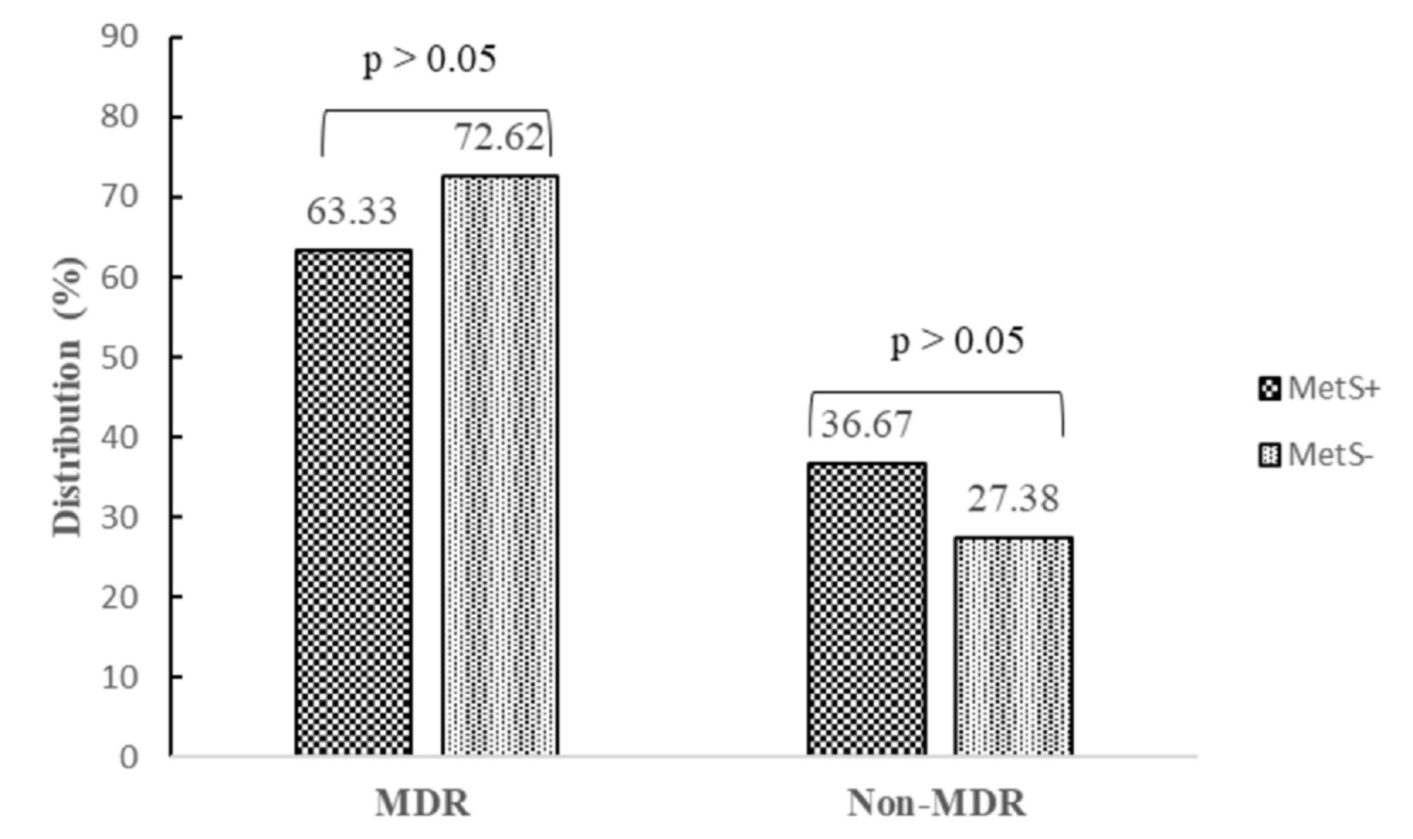
Multidrug-resistant nature of Staphylococcus aureus according to metabolic syndrome status MDR: multidrug-resistant; MetS: metabolic syndrome

The resistances to three and more antibiotics (multidrug-resistant) were screened. Regarding metabolic syndrome status, 72.62% of isolates were multidrug-resistant in non-metabolic syndrome participants versus 63.33% in metabolic syndrome participants (Figure 4).

Table 4 shows the multidrug-resistant nature of *Staphylococcus aureus* among participants with regards to gender and age. Among *Staphylococcus aureus* isolates, 74.60% were multidrug-resistant in females and 64.71% in males. The distribution of multidrug-resistance was significantly different in participants’ age group (p=0.0094, chi-square=9.40). Higher *Staphylococcus aureus* multidrug-resistance was observed in the age group ≥70 years (100.00%), followed by the age group 60 to <70 (78.57%) and 30 to <40 (77.78%).

**Table 4:** Multidrug-resistant nature of Staphylococcus aureus among participants with regards to gender and age MDR: multidrug-resistant; MetS: metabolic syndrome

Variables		Non-MDR (N (%))	MDR (N (%))	X^2^ (probability)
Sex	Male	18 (35.29)	33 (64.71)	1.32 (0.250)
	Female	16 (25.40)	47 (74.60)	
Age group (year)	20 - <30	12 (38.71)	19 (61.29)	9.40 (0.0094)
	30 - <40	2 (22.22)	7 (77.78)	
	40 - <50	11 (40.74)	16 (59.26)	
	50 - <60	6 (31.58)	13 (68.42)	
	60 - <70	3 (21.43)	11 (78.57)	
	≥70	0 (0.00)	14 (100.00)	

## Discussion

The frequency of MRSA in the gastrointestinal tract of metabolic syndrome patients in Mbouda AD LUCEM and District Hospitals was investigated. The prevalence of the *mecA* gene confirming methicillin-resistant Staphylococcus aureus was 79.82% (n=91). The frequency of MRSA was higher in participants with metabolic syndrome (80.00%) as compared to non-metabolic syndrome (79.76%) participants without significant difference (p=0.977). Results also indicated that the frequency of MRSA (91; 79.83%) was higher than that of MSSA (23; 20.17%). The frequency of MRSA in males was 41 (80.39%) and that of females was 50 (79.37%). A high frequency of MRSA was observed in participants aged 60 to <70 and ≥70 years, that is, 92.86% and 100.00%, respectively.

The human intestinal tract harbours a large number of cultivable bacteria such as *Staphylococcus aureus*. The colonization of the gastrointestinal tract by *Staphylococcus aureus* has been documented as potential sources of both endogenous and exogenous staphylococcal infections [5]. In community-acquired MRSA, Beilman et al. have reported that the prevalence rate of MRSA is up to 92%. Kock et al. have reviewed 15 studies and revealed that 13% to 74% of worldwide staphylococcal infections are caused by MRSA [16]. MRSA colonizes body niches as any *Staphylococcus aureus* and the most prevalent site varies between studies as for MSSA.

The antimicrobial susceptibility test revealed *Staphylococcus aureus* isolates had a higher resistance to oxacillin in metabolic syndrome participants as compared to non-metabolic syndrome participants, that is, 100.00% and 92.86%, respectively; non-significant at p=0.322 (chi-square=2.26). The amikacin susceptibility profile was significantly different in metabolic syndrome and non-metabolic syndrome participants (p=0.037, chi-square=6.59). In metabolic syndrome participants, eight (9.52%), 17 (20.24%), and 59 (70.24%) of *Staphylococcus aureus* were resistant,intermediate, and sensitive, respectively, to amikacin versus 0 (0.00%), 12 (40.00%), and 18 (60.00%), respectively, in non-metabolic syndrome participants. In non-metabolic syndrome participants, doxycycline had the highest overall resistance of 85.71%, followed by co-trimoxazole (64.29%) and Chloramphenicol (51.19%) while amikacin had the highest overall sensitivity of 70.24%, followed by gentamicin (64.29%). These results show a marked increase in the resistance of *Staphylococcus aureus* isolates in metabolic syndrome as compared to non-metabolic syndrome participants.

Metabolic syndrome is a disorder of energy use and storage, characterized by central obesity, dyslipidemia, raised blood pressure and high blood sugar levels [17]. Disorders related to the metabolic syndrome could explain a high frequency of resistance of *Staphylococcus aureus* because it has been shown that *Staphylococcus aureus* pathogenesis seems to be closely linked to glucose availability in vitro and in humans [18]. Pomposelli et al. also demonstrated that hospitalized patients who are hyperglycaemic seem to be at a higher risk of *Staphylococcus aureus* infection [19]. 

Antibiotics multidrug-resistance defined as acquired non-susceptibility to at least one agent in three or more antimicrobial categories was studied. In females, 74.60% of *Staphylococcus aureus* isolates were multidrug-resistant, and in males, 64.71%. Of isolates, 72.62% were multidrug-resistant in non-metabolic syndrome participants versus 63.33% in metabolic syndrome participants. Higher *Staphylococcus aureus* multidrug resistance was observed in the age group ≥70 years (100.00%) followed by the age group 60 to <70 (78.57%) and 30 to <40 (77.78%). The explanation for the observed variability may be linked to the differences in the sample size and the antibiotic prescription practices in the study area.

Although this study presents data from metabolic syndrome, patients where information on *Staphylococcus aureus* resistance is extremely limited, we have to acknowledge some limitations. The generalizability of the data might be compromised by sampling biases. In addition, the data should not be generalised to the entire country. We did not collect data in patients below 20 years. In spite of the highlighted limitations, it is still our position that this study presents vital information on the intestinal carriage of MRSA in metabolic syndrome patients. Therefore, the data has an important implication for the quality of patient care and infection control practices.

## Conclusions

This study suggests that metabolic syndrome patients harbour MRSA strains in their intestines even as the difference was not statistically significant with non-metabolic syndrome participants. The need for appropriate antimicrobial use to halt, or at least limit, the spread of resistance is suggested in the care of metabolic syndrome patients and the entire population. The prevention of intestinal carriage of Staphylococcus aureus should be an important clinical concern.
